# A Supramolecular Reinforced Gel Fracturing Fluid with Low Permeability Damage Applied in Deep Reservoir Hydraulic Fracturing

**DOI:** 10.3390/gels10010002

**Published:** 2023-12-20

**Authors:** Yongping Huang, Xinlong Yao, Caili Dai, Yining Wu, Lin Li, Bin Yuan

**Affiliations:** 1 Shandong Key Laboratory of Oilfield Chemistry, China University of Petroleum (East China), Qingdao 266580, China; hypupc@163.com (Y.H.); yaoxl_upc@163.com (X.Y.); wuyining@126.com (Y.W.); 2 School of Petroleum Engineering, China University of Petroleum (East China), Qingdao 266580, China; lilin@upc.edu.cn

**Keywords:** supramolecular reinforced gel, gel fracturing fluid, deep reservoir, gel-breaking solution, low permeability damage

## Abstract

Gel fracturing fluid is the optimum fracturing fluid for proppant suspension, which is commonly applied in deep reservoir hydraulic fracturing. The content of polymers and crosslinkers in gel fracturing fluid is usually high to meet the needs of high-temperature resistance, leading to high costs and reservoir permeability damage caused by incomplete gel-breaking. In this paper, a supramolecular reinforced gel (SRG) fracturing fluid was constructed by strengthening the supramolecular force between polymers. Compared with single network gel (SNG) fracturing fluid, SRG fracturing fluid could possess high elasticity modulus (G′ = 12.20 Pa) at lower polymer (0.4 wt%) and crosslinker (0.1 wt%) concentrations. The final viscosity of SRG fracturing fluid was 72.35 mPa·s, meeting the temperature resistance requirement of gel fracturing fluid at 200 °C. The gel-breaking time could be extended to 90–120 min using an encapsulated gel breaker. Gel particles are formed after the gel fracturing fluid is broken. The median particle size of gel particles in the SRG-breaking solution was 126 nm, which was much smaller than that in the industrial gel (IDG) breaking fluid (587 nm). The damage of the SRG-breaking solution to the core permeability was much less than the IDG-breaking solution. The permeability damage of cores caused by the SRG-breaking solutions was only about half that of IDG-breaking solutions at 1 mD.

## 1. Introduction

The development of conventional oil and gas reservoirs has gradually entered the middle or late period [1,2,3,4]. How to efficiently develop deep reservoirs with abundant reserves attracts the interest of researchers [5,6,7]. The buried depth of the deep reservoir determines that most of its permeability is low, and the process of oil or gas gathering to the bottom of the well will be subject to great flow resistance [8,9]. As a result, the production of the well is low, which makes it difficult to reach the level of economic development. Although some EOR methods, such as asphaltene control and nano flooding, can effectively improve the recovery of low permeability reservoirs, hydraulic fracturing is an important means to realize the efficient development of deep reservoirs [10,11,12]. An instantaneous pressure higher than the formation fracture pressure is generated at the bottom of the well by pumping fracturing fluid into the formation with a high flow rate during hydraulic fracturing, forming many fracturing fractures [13,14]. The fracturing fluids then carry proppant throughout the fracture, maintaining the high permeability of fractures after the pressure is released. The flow resistance of oil or gas can be greatly reduced, and the effective flow area of the oil or gas can be expanded after hydraulic fracturing [15].

Fracturing fluids play an important role in hydraulic fracturing. Their main role is to transfer pressure and carry proppant [16]. Compared with slick water fracturing fluid [17], viscous water fracturing fluid [18], and viscoelastic surfactant fracturing fluid [19,20], gel fracturing fluid is considered to be the strongest water-based fracturing fluid for proppant suspension under high temperatures [21]. Guar gum fracturing fluid is the most widely used among gel fracturing fluids, which is mainly crosslinked using boron crosslinker [22]. High temperatures, some of which can be higher than 180 °C, are common in deep reservoirs. Neither the glucoside bond of the guar gum nor the boron crosslinker can be stabilized at such high temperatures [23,24]. Currently, polyacrylamide-based gels are mainly used to replace guar gum to solve the problem of high-temperature resistance (>180 °C) of gel fracturing fluid. Polyacrylamide-based gels are usually formed by crosslinking a polyacrylamide-based polymer with a transition metal crosslinker (Zr-crosslinker or Ti-crosslinker) [25,26]. Generally, the monomer containing the sulfonic acid group is added to the polyacrylamide to improve the temperature and salt resistance of the polymer, and the cyclic monomer is added to improve the thermal stability of the polymer at high temperatures [27].

Although polyacrylamide-based gels have been able to meet the high-temperature resistance requirements of most fracturing fluids applied in deep reservoirs, the content of polymers and crosslinkers in the gels is generally high in this condition. The high content of polymers and crosslinkers will lead to high costs and incomplete gel-breaking [28]. The permeability damage to the reservoir caused by incomplete gel-breaking will be a problem, which will significantly reduce the effectiveness of deep reservoir fracturing [29,30]. Therefore, it is very important to develop a fracturing fluid with high-temperature resistance and low permeability damage for deep reservoir hydraulic fracturing [31].

Incomplete gel-breaking is due to the high density of crosslinking bonds and tight network structure of gels containing high polymer and crosslinker. Therefore, the key to solving the above problems is to reduce the density of crosslinking bonds without reducing the gel strength. The bond energy of supramolecular forces such as hydrogen bonding, electrostatic interaction, and hydrophobic interaction is much higher than the van der Waals force, which has been widely used in the research and development of hydrogels in recent years [32,33,34]. Therefore, it is feasible to reduce the content of crosslinker by strengthening the supramolecular interaction between polymers to replace some crosslinking bonds [35]. In this paper, a supramolecular reinforced gel fracturing fluid is constructed by introducing functional groups with supramolecular forces into polymers, which possess high-temperature resistance and low reservoir permeability damage after gel-breaking. The research in this paper will provide new insights and theoretical guidance for the development and application of gel fracturing fluids in deep reservoir hydraulic fracturing.

## 2. Results and Discussion

### 2.1. Rheological Properties of Gel Fracturing Fluid

**Figure 1:**
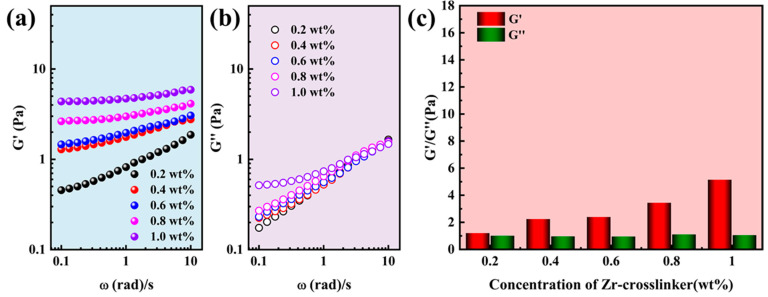
Viscoelasticity of the SNG fracturing fluid (0.4 wt% polymer) at different concentrations of Zr-crosslinker. (**a**) The G′ with frequency. (**b**) The G″ with frequency. (**c**) The G′ and G″ of SNG fracturing fluid at 0.5 Hz.

**Figure 2:**
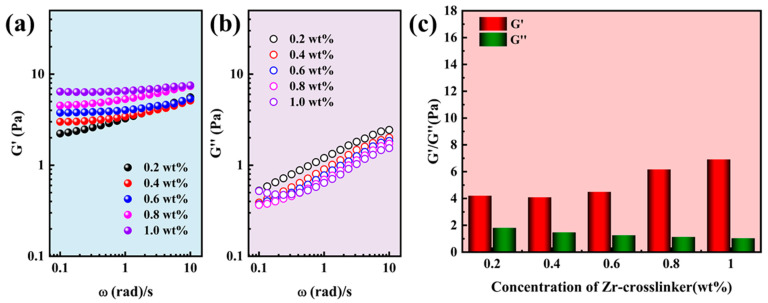
Viscoelasticity of the SNG fracturing fluid (0.6 wt% polymer) at different concentrations of Zr-crosslinker. (**a**) The G′ with frequency. (**b**) The G″ with frequency. (**c**) The G′ and G″ of SNG fracturing fluid at 0.5 Hz.

The main function of gel fracturing fluid is to suspend and transport proppant during the hydraulic fracturing process. However, the high-strength gel fracturing fluid is needed for gel fracturing fluid. The modulus is an important property in judging the strength of the gel fracturing fluid [36,37]. Therefore, the strength of gel fracturing fluid was determined by comparing the modulus of the single network gel (SNG) fracturing fluid and supramolecular reinforced gel (SRG) fracturing fluid. The viscoelastic modulus of SNG (0.4 wt% polymers) fracturing fluid with different concentrations of crosslinkers is shown in Figure 1. The elastic modulus (G′) and viscous modulus (G″) of the SNG fracturing fluid increased with the acceleration of oscillation frequency (Figure 1a,b) [38]. The G′ increased significantly due to the crosslinking network structure of the SNG fracturing fluid becoming tighter with the increase in Zr-crosslinker concentration, but the G″ was unchanged. The G″ was the highest, achieving 5.15 Pa when the concentration of Zr-crosslinker was 1.0 wt% (Figure 1c). Compared to 0.4 wt%, the G′ and G″ were greatly improved when the polymer concentration was increased to 0.6 wt%. Similarly, the G′ and the G″ also increased with the acceleration of the oscillation frequency (Figure 2a,b). Although the G′ of the SNG fracturing fluid increased with the increase in the crosslinker concentration, the G″ decreased. When the concentration of the Zr-crosslinker was 1.0 wt%, the G′ reached 6.91 Pa (Figure 2c).

**Figure 3:**
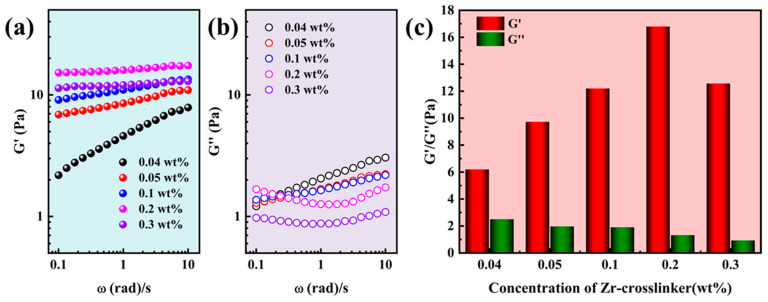
Viscoelasticity of the SRG fracturing fluid (0.4 wt% polymer) at different concentrations of Zr-crosslinker. (**a**) The G′ with frequency. (**b**) The G″ with frequency. (**c**) The G′ and G″ of SRG fracturing fluid at 0.5 Hz.

The G′ and G″ of SRG fracturing fluid are shown in Figure 3. Both the G′ (Figure 3a) and G″ (Figure 3b) increased with the increase in the oscillation frequency, except that the G″ increased at low frequencies when the concentration of Zr-crosslinker was 0.2 wt% and 0.3 wt% [24,25]. Unlike SNG fracturing fluid, SRG fracturing fluid could form high-strength gels at lower Zr-crosslinker concentrations. When the concentration of the crosslinker was 0.04 wt%, the G′ was 6.20 Pa. The G′ reached the maximum (16.80 Pa) at 0.2 wt% Zr-crosslinker and then decreased to 12.57 Pa when the Zr-crosslinker increased to 0.3 wt% [23]. The high G′ of SRG fracturing fluid at a lower Zr-crosslinker concentration was due to the network structure formed by the supramolecular interaction between the polymers; however, whether the shear viscosity could be maintained at a high temperature needed further demonstration.

### 2.2. Shear Resistance of Gel Fracturing Fluid at High Temperatures

**Figure 4:**
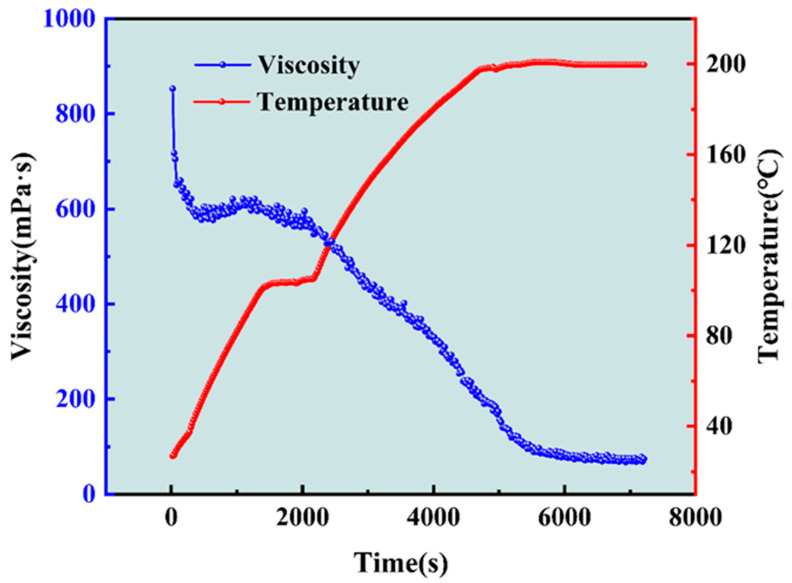
Temperature and shear resistance of SRG fracturing fluid (0.4 wt% polymer + 0.1 wt% Zr-crosslinker). Shear rate: 170 s^−1^.

As could be seen from Figure 3c, when the Zr-crosslinker was 0.1 wt%, the G′ of SRG fracturing fluid reached 12.20 Pa (>10 Pa). Therefore, the concentration of Zr-crosslinker was fixed at 0.1 wt% to conduct the shear experiment at high temperatures, and then it was judged whether the SRG fracturing fluid could meet the high-temperature requirements of deep hydraulic fracturing. The experimental results of shear viscosity at high temperatures are shown in Figure 4. As can be seen from Figure 4, the viscosity stabilized at about 600 mPa·s after the initial shear. During this period, the viscosity did not change with the increase in temperature. When the temperature exceeded 100 °C, the viscosity began to decrease, and the viscosity dropped to 197.8 mPa·s as the temperature reached 200 °C. The viscosity eventually stabilized at 72.35 mPa·s as the shear time continued to 2 h. The criterion of gel fracturing fluid is that the viscosity is greater than 50 mPa·s [25,27]. The final viscosity was sufficient to meet the proppant suspension performance of the gel fracturing fluid, so SRG fracturing fluid fully meets the need for temperature resistance of the gel fracturing fluid applied in deep reservoir hydraulic fracturing.

### 2.3. Gel-Breaking Properties of Gel Fracturing Fluid

**Figure 5:**
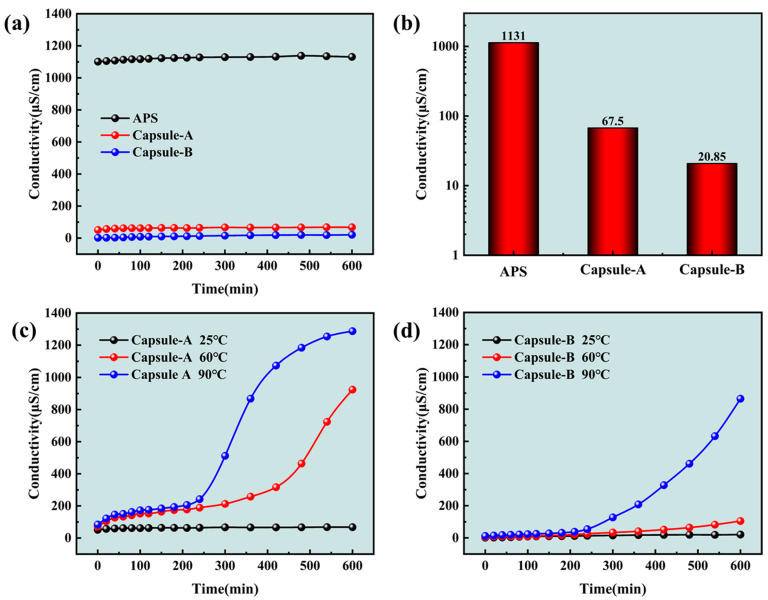
The conductivity of different gel-breaker solutions. (**a**) The conductivity varies with time at 25 °C. (**b**) The conductivity at 600 min (25 °C). (**c**) The conductivity of Capsule-A. (**d**) The conductivity of Capsule-B.

Compared with oxidative-breaking agents such as ammonium persulfate (APS), the encapsulated gel-breaker was beneficial in delaying the rapid degradation of the gel fracturing fluid at high temperatures [39]. The gel-breaker was selected, and the properties of the gel-breaking solution were explored in this section. The conductivity of different gel-breaker solutions is shown in Figure 5. The encapsulated gel-breaker (Capsule-A and Capsule-B) was mainly composed of a gel-breaker wrapped in the capsule. The release of the gel-breaker from the capsule could be judged by the conductivity [40]. It could be seen from Figure 5a that the conductivity of the three gel-breakers (APS, Capsule-A, and Capsule-B) did not change with the increase in soaking time at 25 °C. The conductivities of APS, Capsule-A, and Capsule-B were 1131 μS/cm, 67.5 μS/cm, and 20.85 μS/cm at 600 min, respectively (Figure 5b), indicating that Capsule-A and Capsule-B did not release the gel-breaker. The conductivity of Capsule-A solution at different temperatures is shown in Figure 5c. There was a small increase in conductivity at the initial moment when the temperature was 60 °C or 90 °C. The conductivity increased with the extension of soaking time. A rapid increase in conductivity occurred at 420 min when the temperature was 60 °C, meaning that Capsule-A began to release a large amount of gel-breaker at this moment. The release time was reduced to 240 min when the temperature increased to 90 °C, indicating that the release rate of Capsule-A accelerated with the increase in temperature. The conductivity trend of Capsule-B was similar to that of Capsule-A, as shown in Figure 5d. The difference was that the conductivity of Capsule-B had not a rapid increase in conductivity at 60 °C, and the increased amplitude was much smaller than that of Capsule-A. However, the rapid increase in conductivity also occurred at 240 min when the temperature was increased to 90 °C. From the above experiments, it could be concluded that the conductivity of the encapsulated gel-breaker solution would increase with the increase in time and temperature, which meant that the release rate of the gel-breaker in the capsule was affected by temperature and soaking time. The higher the temperature, the faster the dissolution rate of the surface of the capsule. Therefore, the magnitude of the conductivity is controlled by temperature [41]. Capsule-B has a longer delay time than Capsule-A under the same amount of gel-breaker release. Therefore, Capsule-B was selected as the gel-breaker to conduct the following gel-breaking experiment.

**Figure 6:**
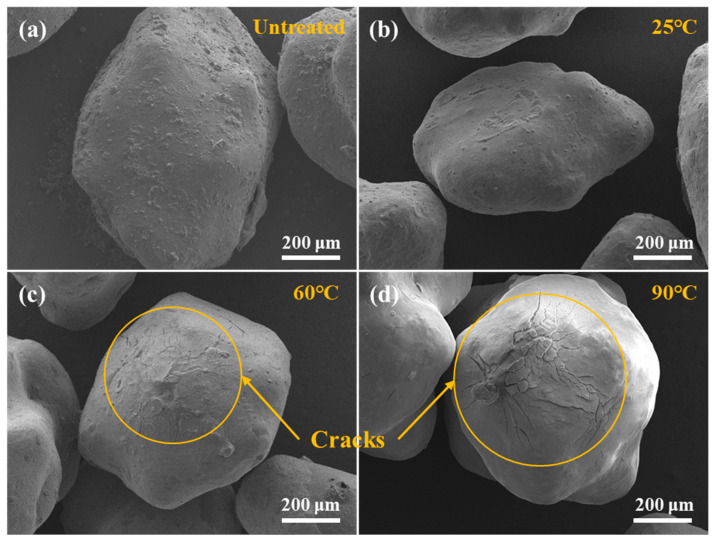
Surface morphology of Capsule-A after being soaked in the water at different temperatures. (**a**) Untreated. (**b**) 25 °C. (**c**) 60 °C. (**d**) 90 °C.

It could be seen from the conductivity experiment that Capsule-A and Capsule-B both had the property of delaying the release of the gel-breaker, but the mode of the delaying release needed further research. The micro-morphology of Capsule-A is shown in Figure 6. The Capsule-A with untreated and soaked in water at 25 °C are shown in Figure 6a,b. No cracks were observed on the surface of the capsules, but the difference was that the surface of the untreated Capsule-A was relatively rough. The reason was that the surface of Capsule-A might be attached to a small amount of gel-breaker, which could also explain why Capsule-A would have a very small conductivity after immersion in water. Some tiny cracks appeared on the surface of Capsule-A when the temperature was 60 °C, as shown in Figure 6c, indicating that Capsule-A began to release the gel-breaker at 60 °C. When the temperature increased to 90 °C, there were more cracks on the surface of Capsule-A, as could be seen in Figure 6d, and the width of the cracks was wider, indicating that the release rate of the gel-breaker should be faster at 90 °C. The above conclusions were consistent with those obtained in Figure 5c. The experiment showed that the surface of Capsule-A would generate cracks after being soaked in high-temperature water and then releasing the gel-breaker [41].

**Figure 7:**
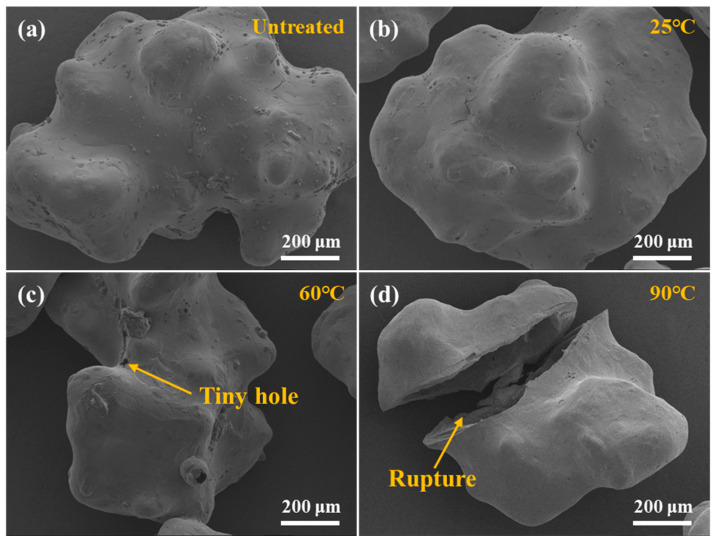
Surface morphology of Capsule-B after being soaked in water at different temperatures. (**a**) Untreated. (**b**) 25 °C. (**c**) 60 °C. (**d**) 90 °C.

The micro-morphology of Capsule-B is shown in Figure 7. There were no obvious cracks or holes on the surface of Capsule-B that was untreated or soaked in 25 °C water, as shown in Figure 7a,b. However, tiny holes were observed when the temperature was 60 °C (Figure 7c). It was found that some Capsule-B even had complete rupture, as shown in Figure 7d, when the temperature increased to 90 °C. It could be concluded from the experiment that the surface of Capsule-B gradually formed holes and might eventually rupture to release the gel-breaker after being soaked in high-temperature water [42].

The breaking time of the SRG fracturing fluid and IDG fracturing fluid under the action of the APS or Capsule-B and the viscosity of the gel-breaking solution are seen in Table 1. It could be seen from the results that the gel-breaking time of the two gel fracturing fluids was about 30–60 min when the gel-breaker was APS. However, when the gel-breaker was Capsule-B, the gel-breaking time of the two gel fracturing fluids was extended to 90–120 min. The gel-breaking time was related to the type of gel-breaker. Compared with APS, Capsule-B had a longer gel-breaking time and could avoid the too-quick degradation of the gel fracturing fluid at 200 °C [43]. The viscosity of all the gel-breaking solutions was less than 2 mPa·s, which could be observed from Table 1.

**Figure 8:**
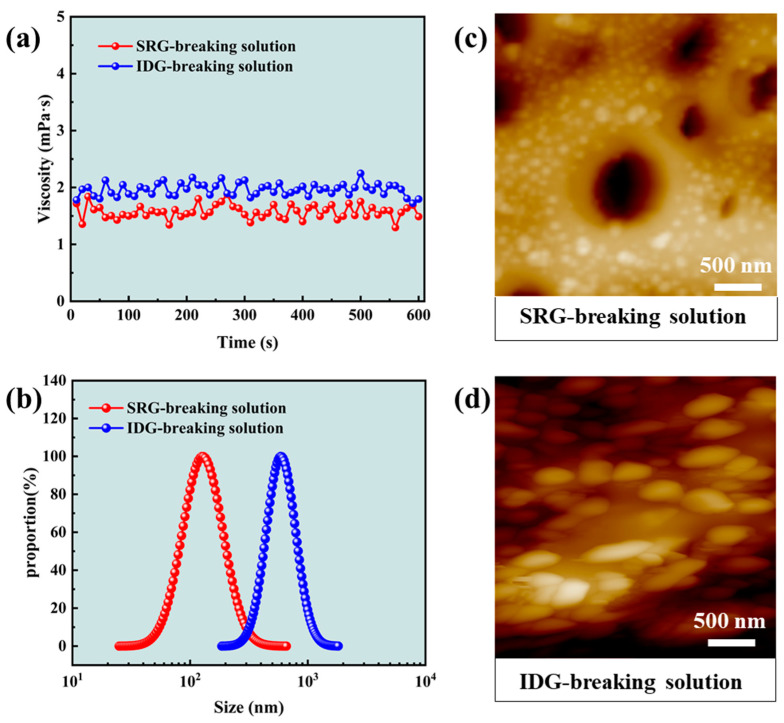
Properties of the gel-breaking solutions. (**a**) Viscosity; (**b**) particle size distribution; (**c**) microtopography of the SRG-breaking solution; (**d**) microtopography of the IDG-breaking solution.

The gel-breaking solutions of the SRG fracturing fluid and industrial gel (IDG) fracturing fluid under the action of Capsule-B were investigated. The viscosity of both gel-breaking solutions was low, as can be seen in Figure 8a, which proves that the gels are all basically broken. However, the particle size of the SRG-breaking solution was much smaller than that of the IDG-breaking solution, as shown in Figure 8b. The distribution size of the SRG-breaking solution and the IDG-breaking solution was 25~658 nm and 186~1814 nm, respectively. The median particle size of the SRG-breaking solution was 126 nm, and the median particle size of the IDG-breaking solution was 587 nm. The difference between the two gel-breaking solutions was 4.66 times. The morphology of the two gel-breaking solutions could be obtained by an atomic force microscope, as shown in Figure 8c,d. The gel particles appeared in the solution after gel-breaking. Compared with the IDG-breaking solution, the particle size of the SRG-breaking solution was smaller, which was consistent with the result in Figure 8b. The above experiments showed that the SRG-breaking solution containing smaller gel particles would be cleaner, which should be conducive to reducing the permeability damage of the reservoir in hydraulic fracturing [44].

### 2.4. Reservoir Permeability Damage under Gel Fracturing Fluid

The damage of different gel-breaking solutions to the permeability of cores was studied according to the diagram of the experimental device shown in 4.2.7, and the results are shown in Table 2. It could be seen from the results that the permeability damage of the cores increased with the decrease in the initial permeability of the two gel-breaking solutions [45]. The permeability damage of 0.1-1, 1-1, and 10-1 was 13.22%, 9.7%, and 7.22%, respectively. The permeability damage of 0.1-2, 1-2, and 10-2 was 26.17%, 16.96%, and 11.04%, respectively. The average permeability damage was 10.05% for the SRG-breaking solution and 18.06% for the IDG-breaking solution. The lower the core permeability, the smaller the pore throat size. The gel particles contained in the gel-breaking solutions would gather in the pore throat and lead to permeability damage. However, the permeability damage of the cores under the SRG-breaking solutions was less than that of the IDG-breaking solutions. The permeability damage of the cores caused by the SRG-breaking solutions was only about half that of the IDG-breaking solutions at 1 mD. The gel particle size in the SRG-breaking solutions was much smaller than that of the IDG-breaking solutions. The larger the gel particle size was, the more likely it was to damage the core permeability [46].

**Figure 9:**
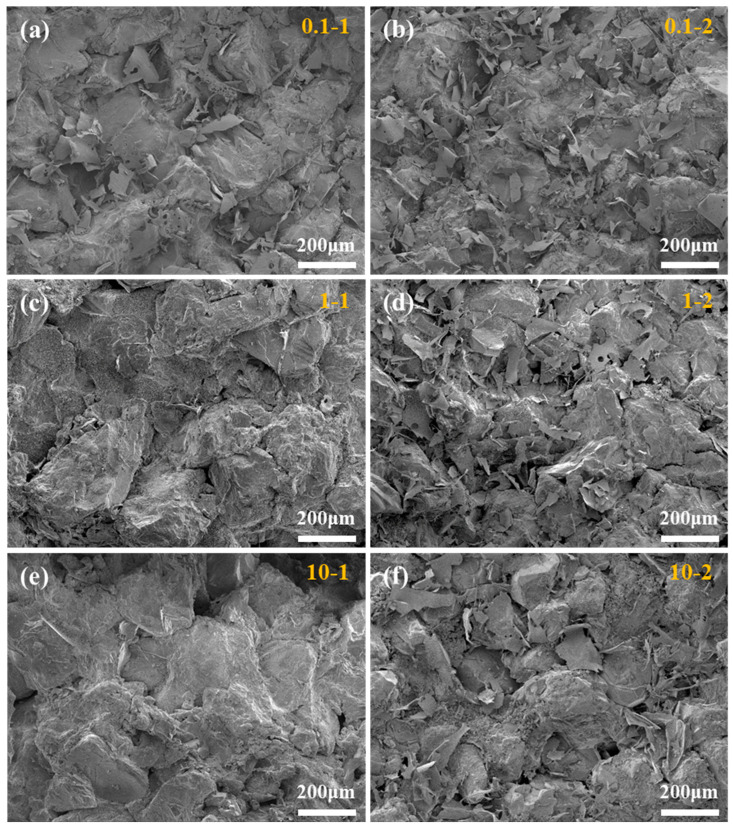
Micromorphology of the different cores after the permeability damage experiment. (**a**) 0.1-1; (**b**) 0.1-2; (**c**) 1-1; (**d**) 1-2; (**e**) 10-1; (**f**) 10-2.

The surface morphology inside the cores after the permeability damage test in Table 2 was observed using SEM, and the results are shown in Figure 9. The surfaces of core 10-1 and core 1-1 were clean, and a small amount of debris could be seen in core 0.1-1, respectively, indicating that the SRG-breaking solution only had obvious damage to core 0.1-1. A certain amount of debris accumulated at the surface of core 0.1-2, core 1-2, and core 10-2, respectively, and more debris was observed with the decrease in core permeability, indicating that the IDG-breaking solution had obvious damage to the permeability of the three cores. It could also be concluded that the damage of the SRG-breaking solution to the core permeability was much lower than that of the IDG-breaking solution. The concentration of the polymer and crosslinker contained in the SRG was lower than that of IDG, and the gel particles after gel-breaking were smaller in size, so there was less damage to the core permeability. Low permeability damage would contribute to the conductivity of the fractured fractures [28].

## 3. Conclusions

In this study, an SRG fracturing fluid was constructed for deep hydraulic fracturing. Compared to the SNG fracturing fluid, the SRG fracturing fluid could obtain higher G′ under lower concentrations of polymer and crosslinker. The network structure formed by the interaction of supramolecular forces between polymers in the SRG replaced the part network structure formed by the crosslinkers. The viscosity of the SRG fracturing fluid after 2 h shearing at 200 °C was 72.35 mPa·s, indicating that it could effectively suspend proppant at 200 °C. Capsule-B did not release the gel-breaker at room temperature. Small holes were gradually formed on the surface of Capsule-B, and some Capsule-B completely ruptured to release the gel-breaker with the increase in temperature. The gel-breaking time of the gel fracturing fluid could be delayed to 90–120 min at 200 °C, avoiding too-quick degradation of gel fracturing fluid. The distribution size of the SRG-breaking solution and the IDG-breaking solution was 25~658 nm and 186~1814 nm, respectively. The median size of the gel particles in the SRG-breaking solution was 126 nm, which was 0.21 times that of the gel particles in the IDG-breaking solution. The smaller particle size of the gel particles in the gel-breaking solution might cause less damage to the reservoir permeability. The permeability damage of cores under the SRG-breaking solution only occurred signally at very low core permeability, and the damage to permeability was much less than that of the IDG-breaking solution. In summary, the SRG-fracturing fluid could maintain the temperature and shear resistance under low polymer and crosslinker concentrations, which also contributed to lower reservoir permeability damage. The novelty of this paper is obtaining a clean solution after the high-strength gel is broken. The gap is that the gel-breaking time is still short at high temperatures, even with the encapsulated gel-breaker.

## 4. Materials and Methods

### 4.1. Materials

The polymers used in this paper are all polyacrylamide-based. Polymer A and polymer B are provided by our laboratory. Chemical enterprises provided industrial polymers and Zr-crosslinkers. Ammonium persulfate (APS) (>98%) was purchased from Sinopharm Group Chemical Reagent Co., LTD, Shanghai, China. Deionized water was provided in our laboratory. Encapsulated gel-breaker A (Capsule-A) was purchased from Dongying Baiyang Petroleum Technology Co., LTD, Dongying, China. Encapsulated gel-breaker B (Capsule-B) was provided by Beijing Shida Ode Technology Co., LTD, Beijing, China. Cores with different permeability were purchased from Beijing Tiandi Kaiyuan Geological Technology Co., LTD, Beijing, China.

### 4.2. Methods

#### 4.2.1. Preparation of the Gel Fracturing Fluids

The composition and shortened form of gel fracturing fluids used in the paper are shown in Table 3. The single network gel (SNG) and industrial gel (IDG) fracturing fluids were formed by adding a Zr-crosslinker to a pre-prepared polymer solution and stirring. To obtain a supramolecular reinforced gel (SRG) fracturing fluid, polymer A and polymer B should be evenly mixed before adding the Zr-crosslinker. The polymer solution was prepared with deionized water.

#### 4.2.2. Rheological Performance Test

All rheological performance tests were conducted using the HAKKE RS600B rheometer (Thermo Hakke Corporation, Waltham, MA, America). A frosted plate module was used for viscoelastic testing at 25 °C to obtain a stable value of the viscoelastic modulus. The viscoelastic modulus of the gel fracturing fluids was obtained using frequency scanning. The shear resistance of the gel fracturing fluids was tested using a drum module. The temperature was gradually raised from 25 °C to 200 °C and then continued to shear for 2 h at 170 s^−1^. Then, 2.75 MPa was pressurized to prevent evaporation before the experiment.

#### 4.2.3. Conductivity Experiment

The three different kinds of gel-breakers were prepared into a 0.1 wt% solution. The solution was placed in ovens at 25 °C, 60 °C, and 90 °C, respectively, and removed at intervals for conductivity measurements. The conductivity of the solution was measured using a conductivity meter (DDS-307, Shanghai Lei Magnetic Instrument Factory, Shanghai, China). The probe of the conductivity meter was thoroughly cleaned with deionized water before each measurement.

#### 4.2.4. Micro-Morphology Experiment

Capsule-A and Capsule-B were immersed in water at 25 °C, 60 °C, and 90 °C for 24 h, respectively. After most of the water was removed, it was frozen with liquid nitrogen for 5 min and then placed in a freeze dryer (Scientz-10N, Ningbo Xinzhi Biotechnology Co., LTD, Ningbo, China) for freeze-drying. The cores of the permeability damage experiment were freeze-dried according to the above steps. The surface structures of the capsules and cores were characterized using scanning electron microscopy (SEM, instrument model: JSM-7610F, magnification: 25–106 times, produced by Nippon Electronics, Showima City, Tokyo, Japan).

#### 4.2.5. Gel-Breaking Test

We added 0.001 wt% APS or 0.001 wt% Capsule-B into the prepared SRG fracturing fluid and IDG fracturing fluid and evenly mixed them in a pressure bottle. The pressure bottle was subsequently placed in the oven at 200 °C, and the time when the gel completely broke was recorded as the breaking time.

#### 4.2.6. Particle Size Measurement of Gel-Breaking Solution

After being set for 24 h, the particle size of the gel-breaking solution was measured using the Nano Brook Omni (Brookhaven Instruments Corporation, New York, NY, America). The experimental temperature was 25 °C.

#### 4.2.7. Permeability Damage Experiment

**Figure 10:**
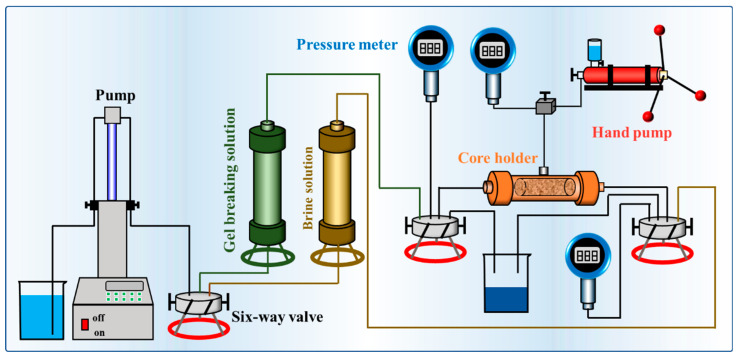
Schematic diagram of permeability damage experiment.

Six cores with different permeability (Table 2) were used in the reservoir damage experiment, and the experimental diagram is shown in Figure 10. The green tank contained the gel-breaking solution, and the yellow tank contained the brine solution (2.0 wt% KCl + 5.5 wt% NaCl + 0.45 wt% MgCl_2_ + 0.55 wt% CaCl_2_). We loaded the core into the core holder and adjusted the confining pressure to 2.5 MPa. The brine solution was reversely injected into the core at 0.1 mL/min flow rate to obtain a stable injection pressure *P*_1_. Then, the gel-breaking solution was injected, and the pump was stopped to stabilize for 2 h. Finally, the brine solution was reversely injected into the core at 0.1 mL/min flow rate to obtain a stable injection pressure *P*_2_. The core permeability *K*_1_ and *K*_2_ corresponding to injection pressure *P*_1_ and *P*_2_ were calculated using Darcy’s law, respectively. The formula for calculating the reservoir permeability damage was as follows:

(1) $$\eta =\frac{K_{1}-K_{2}}{K_{1}}\times 100\%$$

## Figures and Tables

**Table 1:** Gel-breaking time of gel fracturing fluid under different gel-breakers.

Gel Fracturing Fluid	Gel-Breaker	Gel-Breaking Time/min	The Viscosity of Gel-Breaking Solution/mPa·s
SRG	APS	30–60	1.28
SRG	Capsule-B	90–120	1.57
IDG	APS	30–60	1.71
IDG	Capsule-B	90–120	1.96

**Table 2:** Permeability damage under different gel-breaking solutions.

Core Label	Gel-Breaking Solution	Permeability K_1_/mD	Permeability K_2_/mD	Permeability Damage, η/%
0.1-1	SRG	0.121	0.105	13.22
1-1	SRG	1.691	1.527	9.70
10-1	SRG	10.983	10.190	7.22
0.1-2	IDG	0.107	0.079	26.17
1-2	IDG	1.834	1.523	16.96
10-0	IDG	9.511	8.461	11.04

**Table 3:** The composition and shortened form of the gel fracturing fluids.

Gel Type	Polymer	Crosslinker	Shortened Form
Supramolecular reinforced gel	polymer A: polymer B = 2:1	Zr	SRG
Single network gel	Polymer A	Zr	SNG
Industrial gel	Industrial polymer	Zr	IDG

## Data Availability

The data presented in this study are openly available in article.
